# Lactic Acid Fermentation of *Arthrospira platensis* (Spirulina) in a Vegetal Soybean Drink for Developing New Functional Lactose-Free Beverages

**DOI:** 10.3389/fmicb.2020.560684

**Published:** 2020-10-26

**Authors:** Alberto Niccolai, Kaja Bažec, Liliana Rodolfi, Natascia Biondi, Emil Zlatić, Polona Jamnik, Mario R. Tredici

**Affiliations:** ^1^Department of Agriculture, Food, Environment and Forestry, University of Florence, Florence, Italy; ^2^Department of Food Science and Technology, Biotechnical Faculty, University of Ljubljana, Ljubljana, Slovenia; ^3^Fotosintetica & Microbiologica S.r.l., Florence, Italy

**Keywords:** lactic acid fermentation, spirulina, vegetal soybean drink, proteins, antioxidants, digestibility, lactose-free functional beverage

## Abstract

The main objective of this study was to evaluate the suitability of *Arthrospira platensis* F&M-C256 (spirulina) biomass in a vegetal soybean drink or in water, as substrate for lactic acid fermentation by the probiotic bacterium *Lactiplantibacillus plantarum* ATCC 8014 (LAB8014) and to evaluate the fermented products in terms of bacteria content and organic acids content, biochemical composition, total phenolics, and phycocyanin content, *in vitro* digestibility, *in vitro* and *in vivo* antioxidant activity. After 72 h of fermentation, a bacterial concentration of about 10.5 log CFU mL^–1^ in the broths containing the soybean drink + spirulina + LAB8014 (SD + S + LAB8014) or water + spirulina + LAB8014 (W + S + LAB8014) was found. Lactic acid concentration reached similar values (about 1.7 g L^–1^) in the two broths, while a different acetic acid concentration between SD + S + LAB8014 and W + S + LAB8014 broths was observed (7.7 and 4.1 g L^–1^, respectively). *A. platensis* biomass was shown to be a suitable substrate for LAB8014 growth. After fermentation, both broths contained a high protein content (>50%). In both broths, total phenolics, *in vitro* and *in vivo* antioxidant activity increased after fermentation (+35, +20, and +93% on average, respectively), while phycocyanin content decreased (−40% on average). Digestibility of W + S + LAB8014 broth statistically improved after fermentation. This study highlights the potential of *A. platensis* F&M-C256 biomass as a substrate for the production of new functional lactose-free beverages.

## Introduction

Lactic acid fermentation is widely used as a food preservation method and to ameliorate the aroma of food and beverages exploiting the ability of lactic acid bacteria (LAB) to produce volatile compounds during fermentation (Smid and Kleerebezem, 2014; de Marco Castro et al., 2019). LAB can also improve the texture and nutraceutical profile of foods, beverages, and of by-products derived from food processing (Ricci et al., 2019a,c; Sozer et al., 2019). Lactic acid fermentation can be considered a valuable technology to enhance safety, shelf life, sensory and nutritional properties of vegetables and fruits (Di Cagno et al., 2013; Ricci et al., 2019b; Muhialdin et al., 2020; Thompson et al., 2020). Several studies on LAB showed their: (i) capacity to resist intact to the transit through the gastric tract, (ii) ability to colonize the host gut, and (iii) safety and potential health benefits such as cholesterol reduction (de Vries et al., 2006; Karczewski et al., 2010). LAB during fermentation probably degrade cyanobacterial cell walls, via the wide array of peptidoglycan hydrolyses, resulting in the extraction and conversion of complex organic compounds (such as polysaccharides, lipids, and proteins) within the cell, into smaller molecules with antioxidant, immunomodulatory, and anti-inflammatory activity (Frirdich and Gaynor, 2013; de Marco Castro et al., 2019).

The request for dairy-free alternatives is rapidly increasing because of the rising incidence of lactose intolerance and veganism (Di Cagno et al., 2013). Recently, increased demand for fortified food and beverage products with higher nutritional quality from sportsmen, elderly, and children was also observed (Di Cagno et al., 2013; de Marco Castro et al., 2019). Some algae represent a suitable substrate for the production of probiotic lactose-free foods and beverages through lactic acid fermentation due to their high nutritional value and/or valuable components (Gupta et al., 2011; Ścieszka and Klewicka, 2019; Martelli et al., 2020a), e.g., polysaccharides from algae are already recognized and accepted as dietary prebiotics (Jiménez-Escrig et al., 2001; de Jesus Raposo et al., 2016).

A previous study demonstrated the suitability of the cyanobacterium *Arthrospira platensis* F&M-C256 as substrate for *Lactiplantibacillus plantar*um ATCC 8014 (LAB8014) growth (Niccolai et al., 2019a). *A. platensis*, commercially known as spirulina, is a source of macro and micronutrients including proteins, iron, γ-linolenic acid, vitamins, minerals, phycocyanin, and sulfated polysaccharides (Kulshreshtha et al., 2008; Liu et al., 2011). *Arthrospira* species also show several activities of pharmacological interest such as immunomodulatory, lipid-lowering, anti-inflammatory, and antioxidant effects (Colla et al., 2008; Kim et al., 2010; Gutiérrez-Rebolledo et al., 2015; Bigagli et al., 2017) and no *in vitro*/*in vivo* adverse effects were found (Bigagli et al., 2017; Niccolai et al., 2017). The safety of *Arthrospira* for human consumption is supported by its long history of use as food ingredient (Abdulqader et al., 2000). *A. platensis* is considered safe in many countries (e.g., in EU, Australia, United States) and widely commercialized as dietary supplement (powder, capsules, or tablets) (Borowitzka, 2013). *A. platensis* biomass is also increasingly used as food ingredient, incorporated into gluten-free pasta (Fradinho et al., 2020), bakery products (Batista et al., 2017, 2019; Niccolai et al., 2019b), candies, yogurt, and soft drinks (Christaki et al., 2011; Niccolai et al., 2019a).

Several authors (de Caire et al., 2000; Varga et al., 2002; Bhowmik et al., 2009; Guldas and Irkin, 2010; Beheshtipour et al., 2013; Mazinani et al., 2016; Yamaguchi et al., 2019; Martelli et al., 2020b) evaluated the effect of *Arthrospira* spp. biomass addition to yogurt, cheese, and fermented milk showing an increase in the number of LAB and improvement of the nutritional quality of the products during storage. Fermented products, powders, beverages, or delicacies from seaweeds, *Chlorella*, *Dunaliella*, and spirulina (different *Arthrospira* species), usually mixed with plant-derived substrates and obtained through fermentative processes by LAB or yeasts or a mixture of these microorganisms, are already available on the market (de Marco Castro et al., 2019). To the best of our knowledge, except for the work of Martelli et al. (2020b), no work concerning the effect of *A. platensis* fermentation in non-dairy beverages, specifically in a soybean drink, as well as on *in vivo* intracellular oxidation and phycocyanin (PC) and allophycocyanin (APC) stability was carried out.

The aim of this study was to investigate the suitability of *A. platensis* F&M-C256 biomass as substrate for LAB8014 growth and fermentation in a vegetal soybean drink or in water and to evaluate the fermented product in terms of bacteria content, *in vitro* digestibility, and *in vitro* and *in vivo* antioxidant capacity, which are parameters of great importance as first steps for the development of new lactose-free functional beverages.

## Materials and Methods

### *Arthrospira platensis* F&M-C256 Biomass Production

*Arthrospira platensis* F&M-C256 belongs to the Fotosintetica & Microbiologica S.r.l. Microalgae Culture Collection. The cyanobacterial biomass was produced, as reported in Niccolai et al. (2019a) at Azienda Agricola Serenissima S.S. (Padova, Italy). The cyanobacterium was cultivated in Zarrouk medium (Zarrouk, 1966), in semi-batch regime, in GWP^®^-II photobioreactors (Tredici et al., 2016). The biomass was harvested by filtration and washed with tap water to remove excess bicarbonate, dried at 33°C (North West Technology, Italy) for 20 h and then stored at −20°C until use. The biochemical composition of the biomass was determined as reported in Abiusi et al. (2014) (Table 1).

**TABLE 1:** Biochemical composition of *A. platensis* F&M-C256 biomass used in the experiments.

	Moisture	Ash	Lipid	Protein	Carbohydrate
*A. platensis* F&M-C256	7.7 ± 0.1	5.8 ± 0.1	6.7 ± 1.3	67.3 ± 0.5	12.5 ± 0.2

*Data are expressed as % of algal powder. Values are expressed as mean ± s.d.*

### *Lactiplantibacillus plantarum* Inoculum Preparation

*Lactiplantibacillus plantarum* ATCC 8014 (LAB8014) was obtained from Cruinn Diagnostics Ltd. (Ireland). The LAB8014 cultures were maintained at −80°C in glycerol (20%) stocks. Twenty-five milliliters of autoclaved MRS broth (Scharlau Chemie, Spain) were inoculated with 1 mL of defrosted LAB8014 stock culture. The culture was revitalized through 2–3 steps of growth and dilution. The cultures were incubated at 37°C for one day in an orbital shaker (Gallenkamp, Weiss Technik, United Kingdom). After revitalization, the inoculum was serially diluted 100 times to obtain a working culture containing 8–9 log CFU mL^–1^ as determined by plate counts following Niccolai et al. (2019a).

### Lactic Acid Fermentation

**FIGURE 1:**
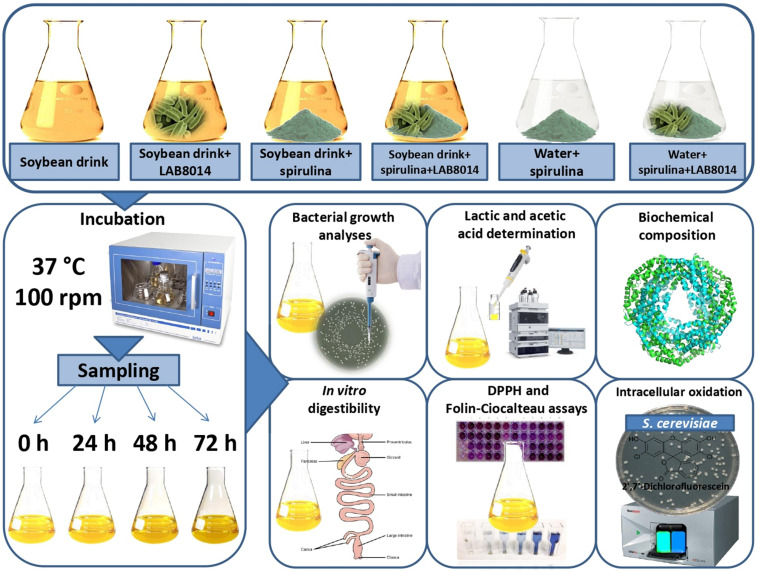
Plan of the experiments.

The evaluation of *A. platensis* F&M-C256 biomass as substrate for LAB8014 growth and fermentation was assessed according to Niccolai et al. (2019a). Five grams of lyophilised *A. platensis* F&M-C256 biomass (S) (with 8% of residual water content) was introduced, under sterile conditions, in a 100 mL Erlenmeyer flask and 40 mL of commercial vegetal soybean drink (SD) (Alce Nero S.p.a., Italy) or water (W) was added. The biochemical composition of the vegetal drink is presented in Table 2. The vegetal drink contains soybeans (7.5%) and the red seaweed *Lithothamnium calcareum* (0.4%). The suspensions were inoculated with 41 μL of a LAB8014 culture (hereafter: SD + S + LAB8014 and W + S + LAB8014, respectively). Vegetal soybean drink or water was then added up to the final volume of 50 mL. Thus, the initial *A. platensis* F&M-C256 biomass concentration in the inoculated suspension was 92 g dry weight L^–1^. A broth containing the vegetal soybean drink and LAB8014 (41 μL), with a final volume of 50 mL (hereafter: SD + LAB8014) was prepared. Broths containing soybean drink and *A. platensis* F&M-C256 biomass, water and *A. platensis* F&M-C256 biomass, and only soybean drink were also prepared (hereafter: SD + S, W + S, and SD, respectively). The experimental design is reported in Figure 1. The flasks (three replicates) were incubated at 37°C and 100 rpm. Samples were taken at 0 (immediately after inoculation), 24, 48, and 72 h in triplicate for microbiological and chemical analyses and for pH measurement following Niccolai et al. (2019a). According to Gupta et al. (2011), a period of 72 h was considered adequate to observe and evaluate the principal fermentation parameters. To evaluate *in vitro* digestibility (estimated at 0 and after 72 h of incubation), phycocyanin content (only for *A. platensis* F&M-C256-based broths), phenolics, and *in vitro* and *in vivo* antioxidant capacity (after 0, 24, 48, and 72 h of incubation) aliquots in triplicate (15 mL) were taken and lyophilised before analysis.

**TABLE 2:** Nutritional declaration of commercial vegetal soybean drink “Alce Nero” used in the experiments.

	Lipid (g)	Protein (g)	Carbohydrate (g)	Salt (g)	Calcium (mg)	Energy (Kcal)
Vegetal soybean drink	1.8	3	1	0.1	120	34

*Values are expressed per 100 mL of product.*

### Microbiological Analyses

Cultivable bacterial cell counts in the broths, expressed as log CFU mL^–1^, were carried out in triplicate at the start (0 h) and after 24, 48, and 72 h, by plating on MRS agar (Scharlau Chemie, Spain). The fermentation broths were serially diluted (1:10) in a saline solution (0.9% w/v NaCl) before plating, following the procedure reported by Niccolai et al. (2019a). The plates were then incubated at 37°C for 36–48 h.

### Organic Acids Determination

The concentration of lactic and acetic acid was determined by HPLC. The cell-free supernatant of fermented broth was diluted 1:10 with 5 mM H_2_SO4 (Sigma-Aldrich, Germany) and filtered through a 0.22 μm PTFE syringe filter (Macherey-Nagel, Germany) prior to HPLC injection. The organic acids were analyzed on Agilent 1260 Infinity HPLC system (Agilent Technologies, California, CA, Untied States), equipped with a diode array detector (DAD); the wavelength was set at 210 nm. Ten microliters of the diluted samples were injected onto an Aminex HPX-87H (300 mm × 7.8 mm) column (Bio-Rad, Hercules, CA, United States). The compounds were eluted at 60°C with an isocratic flow rate of 0.6 mL/min using 5 mM H_2_SO4 in water as mobile phase. The data acquisition and integration were performed using the MassHunter^TM^ 6.0 software package. Each sample was injected twice. Commercial standards of organic acids (Sigma-Aldrich, Germany) were used to identify and quantify lactic and acetic acid in the samples.

### Biochemical Composition

Lyophilised broths were analyzed for total protein, carbohydrate, lipid, moisture, and ash content. Total protein was analyzed following Lowry et al. (1951). Carbohydrate and lipid were quantified according to Dubois et al. (1956) and Marsh and Weinstein (1966), respectively. Moisture and ash were determined following ISTISAN protocols (ISTISAN Report, 1966).

### *In vitro* Digestibility

The *in vitro* digestibility was evaluated by the method of Boisen and Fernández (1997), modified by Niccolai et al. (2019c), on lyophilised fermentation broths at the start (time 0) and at the end (72 h) of fermentation. The *in vitro* process simulates the chemical-enzymatic attack, by gastric and pancreatic juices, that takes place in the terminal part of the digestive system of the monogastric apparatus. The method consists of a double enzymatic incubation, first by porcine pepsin (Applichem, Germany) and then by porcine pancreatin (Applichem, Germany). At the end of fermentation (72 h), broths samples were collected, lyophilised and powdered before analysis. One-gram samples (particle size ≤1 mm) were prepared for analysis in 250 mL conical flasks.

### Phycocyanin, Phenolics, and Antioxidant Activity Determination

Phycocyanin content was assessed according to Herrera et al. (1989) on samples of lyophilised fermentation spirulina-based broths (1 g) at times 0, 24, 48, and after 72 h of fermentation. The method is based on the extraction of this water soluble pigment with calcium chloride (1% w/v) at pH 6.8 (20°C) and consequent spectrophotometric quantification at 620 nm and 650 using a UV-Vis spectrophotometric reader (Cary 60 UV-Vis, Agilent Technologies, California, CA, United States).

Phenolics were quantified according to Ganesan et al. (2008). Lyophilised samples (0.1 g) of broths at time 0 and after 24, 48, and 72 h of fermentation were dissolved in deionised water (10 mL). To 100 μL aliquots of each sample, sodium carbonate (2 mL at 2% in water) (Sigma-Aldrich) was added. After 2 min, 100 μL of a Folin Ciocalteu:water (1:1) solution (Sigma-Aldrich, Germany) was added. The mixture was let to react in the dark at 25°C for 30 min. The absorbance was measured at 720 nm using a UV-Vis spectrophotometric reader (Cary 60 UV-Vis, Agilent Technologies, California, CA, United States). Results were expressed in mg of gallic acid equivalents per g of sample (mg GAE g^–1^) using a calibration curve built with gallic acid (0–500 μg mL^–1^) (Sigma-Aldrich, Germany).

The radical scavenging ability of the fermentation broth at times 0, 24, 48, and 72 h was assessed by the 2,2-diphenyl-1-picrylhydrazyl (DPPH) radical test (Sigma-Aldrich, Germany). The test was carried out with 100 μL of DPPH solution (165 μM in methanol) and 100 μL of sample (0.2 g of lyophilised broths extracted for 30 min in 5 mL of a 1:5 methanol:water solution) according to Rajauria et al. (2013). The reaction was incubated in the dark at 30°C for 30 min. The absorbance was measured at 517 nm by means of a UV-Vis spectrophotometer (Cary 60 UV-Vis, Agilent Technologies, California, CA, United States).

### Determination of Intracellular Oxidation

#### Yeast Strain and Cultivation

The yeast *Saccharomyces cerevisiae* ZIM 2155 was obtained from the Culture Collection of Industrial Microorganisms (ZIM) of the Biotechnical Faculty (University of Ljubljana, Slovenia). The yeast cells were cultivated in YEPD medium at 28°C and 220 rpm until the stationary phase. The yeast culture was then centrifuged for 3 min at 14,000 rpm, washed once with phosphate-buffered saline (PBS) (Merck KGaA, Germany) and cells were resuspended in PBS at a concentration of 8 log cells mL^–1^. Further 96-h incubation at 28°C and 220 rpm was carried out.

#### Lyophilised Fermented and Non-fermented Broths Water Extracts Preparation

One gram of the lyophilised broths was suspended in 9.5 mL of sterile water and sonicated for 20 min in ice, in order to maintain the temperature below 10°C. Then, the probe of the sonicator was washed with 0.5 mL of sterile water and the supernatant was recovered after centrifugation at 6,000 rpm for 60 min. To remove any possible debris, a second centrifugation at 6,000 rpm for 30 min on supernatant was carried out. An aliquot (0.5 mL) was dried at 80°C for 24 h to determine the extract dry weight. A water extract from the lyophlised *A. platensis* F&M-C256 biomass, following the same procedure, was also prepared. The extracts were set to the same concentration of dry weight (32 mg mL^–1^) and stored at −20°C until use.

#### Treatment of Yeast Cells

The water extracts prepared from lyophilized *A. platensis* F&M-C256 biomass and from lyophilised broths were added to the yeast cell suspensions following 96-h incubation in PBS, at 50 μL extract mL^–1^ of yeast cell suspension. To measure intracellular oxidation the samples were taken after a further 2-h incubation of yeast suspension at 28 °C and 220 rpm.

#### Determination of Intracellular Oxidation

Intracellular oxidation was determined by using 2′,7′-dichlorofluorescein (H_2_DCF) (Sigma-Aldrich, Germany), which reacts with oxidants, thus revealing the presence of reactive oxygen species (ROS). The dye was added to the cells as 2′,7′-dichlorofluorescein diacetate (H_2_DCFDA), which easily enters the cells and it is hydrolysed by non-specific cellular esterases. H_2_DCF can then be oxidized to fluorescent 2′,7′-dichlorofluorescin (DCF) by intracellular ROS, which is detected fluorimetrically (Jakubowski and Bartosz, 1997). From 10 mL suspension containing yeast cells treated with extracts, 2 mL of the suspension was taken, washed with 50 mM potassium phosphate buffer at pH 7.8 and centrifuged at 14,000 rpm for 5 min. Finally, the cell pellets were resuspended in 500 μL of 50 mM potassium phosphate buffer. Then 100 μL of suspension was transferred to 890 μL of 50 mM potassium phosphate buffer and incubated (28°C, 10 min). Ten microliters of H_2_DCFDA (Sigma-Aldrich, Germany) (1 mM stock solution) was added to the mixture followed by 30-min incubation at 28°C and 220 rpm. Then, cells were sedimented by centrifugation (14,000 rpm, 5 min) and resuspended in 1 mL of fresh 50 mM potassium phosphate buffer to measure the fluorescence using a Varioskanr^TM^ LUX Multimode Microplate Reader (Thermo Fisher Scientific, Massachusetts, MA, United States). The excitation and emission wavelengths of DCF were 488 and 520 nm, respectively. The OD of yeast suspension was measured at 650 nm to normalize data of fluorescence. Results are expressed as means of relative values (fluorescence intensity/optical density, F/OD) ± SD normalized to the control (non-treated yeast cells).

### Statistical Analysis

The analyses were carried out in triplicate on three broths. The results were expressed as mean ± SD (standard deviation). All statistical analyses were performed using Statgraphics Centurion XV (StatPoint Technologies Inc., Washington, DC, United States). Statistical differences between different broths were determined using ANOVA followed by the Duncan’s Multiple Range Tests (MRT) to determine the Least Significant Difference (LSD). Differences were considered significant when *P* < 0.05.

## Results

### Suitability of *A. platensis* F&M-C256 Biomass for *L. plantarum* ATCC 8014 Growth

**FIGURE 2:**
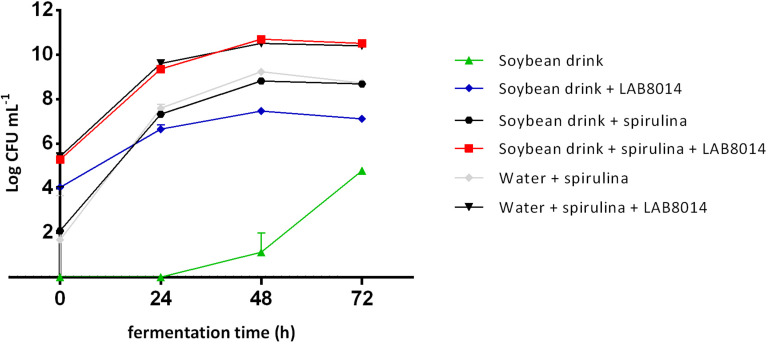
Bacterial growth curves in the different broths. Values are expressed as mean ± s.d. The amount of *A. platensis* F&M-C256 biomass at the start of the experiment (0 h) for the spirulina-based broths was 92 g (dry weight) L^– 1^.

The bacterial growth curves, expressed as log CFU per mL of broth, are shown in Figure 2. The initial bacterial load associated with spirulina biomass is on average 1.9 ± 0.3 log CFU mL^–1^, that is about three orders of magnitude lower than SD + S + LAB8014 and W + S + LAB8014 broths (5.4 ± 0.1 log CFU mL^–1^ on average). At the start of fermentation (0 h), SD + LAB8014 broth showed a bacterial concentration of 4.0 ± 0.1 log CFU mL^–1^. With the exception of SD broth, the highest bacterial concentrations were reached after 48 h of fermentation, while later a decrease in the microbial concentration was observed.

Soybean drink showed to be a good substrate for LAB8014 growth (7.5 log CFU mL^–1^ for SD + LAB8014 broth after 48 h). *A. platensis* F&M-C256 demonstrated to be an even better substrate for LAB8014 (10.5 log CFU mL^–1^ for W + S + LAB8014 broth after 48 h) as well as for its associated bacteria (9.2 log CFU mL^–1^ for W + S broths). The combination of the two substrates gave the best results in terms of LAB8014 growth (10.7 log CFU mL^–1^ for SD + S + LAB8014 broth after 48 h), while a reduction in bacteria associated with *A. platensis* F&M-C256 biomass (8.8 log CFU mL^–1^ for SD + S broth after 48 h) was observed. The MRS medium mainly sustains LAB growth. Preliminary tests (not reported in this study) were performed on the bacteria associated with *A. platensis* F&M-C256 biomass. Growth on MRS agar in microaerophilic condition was compared with that on Nutrient Agar in microaerophilic and aerobic conditions. No relevant differences between the tested conditions after 24 h of incubation were observed.

The optimal substrate for LAB8014 growth was SD + S. Maximal bacterial concentration in SD + S+LAB8014 broth was 10.7 ± 0.1 log CFU mL^–1^, corresponding to 11.7 log CFU per g of *A. platensis* F&M-C256 dry biomass initially added (92 g L^–1^). Considering the average weight of a LAB8014 cell (0.48 ± 0.07 pg) (Niccolai et al., 2019a), the amount of LAB8014 after 48 h was 0.024 g mL^–1^ and the yield of bacterial biomass from *A. platensis* F&M-C256 was 26.1%. In W + S + LAB8014 (without SD nutrients) after 48 h of fermentation bacterial concentration was 10.5 log CFU mL^–1^, equivalent to 0.015 g mL^–1^, with a yield on *A. platensis* F&M-C256 biomass of 16.5%. It has to be noted that in the case of SD + S + LAB8014 the bacterial biomass derives not only from *A. platensis* F&M-C256 biomass but also from nutrients in SD.

The bacterial concentration in the other broths were one in SD + S and W + S or three in SD + LAB8014 orders of magnitude lower than in SD + S+LAB8014 and in W + S + LAB8014 broths. As expected, SD not inoculated with LAB8014 contained a very low number of bacteria, not detectable from the start (0 h) up to 24 h of fermentation. The bacterial growth started after 24 h, reaching 1.1 ± 0.85 log CFU mL^–1^ after 48 h and showed the highest concentration at 72 h of fermentation (4.8 ± 0.13 log CFU mL^–1^).

At the start of fermentation, SD and SD + LAB8014 broths showed pH values of 8.2 and 8.3, respectively. The addition of *A. platensis* F&M-C256 acidified the broths (pH 6.4 on average). The W + S broth showed the highest pH decrease compared to the other spirulina-based broths. Overall, an average decrease of pH (−8%) from the start to the end of fermentation for all the analyzed broths was observed (Table 3).

**TABLE 3:** Trend of pH of the different broths during fermentation.

	Fermentation times (h)			
	
	0	24	48	72
Soybean drink	8.22 ± 0.03^*a*^	6.88 ± 0.03^*c*^	6.82 ± 0.09^*c*^	7.59 ± 0.04^*b*^
Soybean drink + LAB8014	8.30 ± 0.02^*a*^	6.79 ± 0.02^*c*^	6.68 ± 0.06^*d*^	6.99 ± 0.09^*b*^
Soybean drink + spirulina	6.63 ± 0.02^*a*^	6.08 ± 0.02^*b,c*^	6.04 ± 0.02^*c*^	6.12 ± 0.04^*b*^
Soybean drink + spirulina + LAB8014	6.47 ± 0.03^*a*^	5.99 ± 0.04^*b*^	5.71 ± 0.03^*b*^	5.94 ± 0.02^*c*^
Water + spirulina	6.13 ± 0.01^*a*^	5.85 ± 0.03^*b*^	5.58 ± 0.03^*c*^	5.58 ± 0.02^*c*^
Water + spirulina + LAB8014	6.50 ± 0.02^*a*^	5.57 ± 0.03^*c*^	5.53 ± 0.03^*c*^	5.93 ± 0.08^*b*^

*Values are expressed as mean ± s.d. Different letters in a row denote a significant difference (*P* < 0.05).*

**FIGURE 3:**
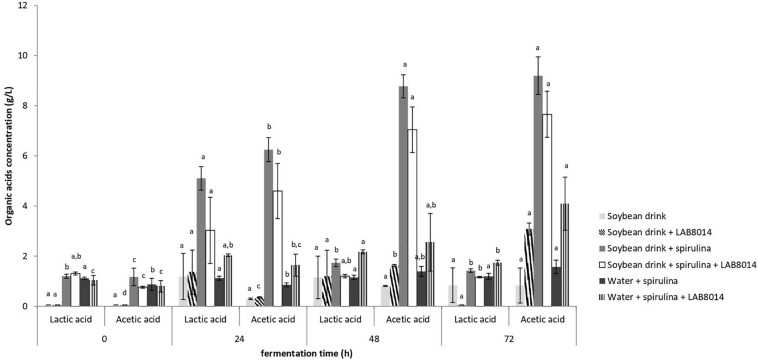
Lactic and acetic acid production by LAB8014 in the different fermentation broths. Values are expressed as mean ± s.d. Different letters between columns of the same broth combination, for lactic or acetic acid, show significant differences (*P* < 0.05).

Lactic and acetic acid in the broths during fermentation is reported in Figure 3. The highest amount of lactic acid was found after 24 h of fermentation in all the broths. SD + S and SD + S + LAB8014 broths accumulated up to 5.1 and 3 g L^–1^ of lactic acid with a conversion yield of 55 and 33 mg of lactic acid per gram of dry *A. platensis* F&M-C256 biomass, respectively. A lower amount (2 g L^–1^) was detected in W + S + LAB8014 broth. Concerning acetic acid, the highest concentration was found after 48 and 72 h of fermentation in all the analyzed broths. After 72 h of fermentation, SD + S and SD + S + LAB8014 broths accumulated up to 9.2 and 7.7 g L^–1^ of acetic acid with a conversion yield of 100 and 84 mg of acetic acid per gram of dry *A. platensis* F&M-C256 biomass, respectively. As expected a lower concentration (4.1 g L^–1^) of acetic acid was detected in W + S + LAB8014 broth, considering the absence in water of the nutrients available for bacterial growth in the soybean drink (Table 2).

The biochemical composition of the different lyophilised broths is shown in Table 4. As expected, carbohydrates were consumed by bacteria as energy source during fermentation. In all the broths, after 72 h of fermentation, a significant average reduction (*P* < 0.05) of about 30% in carbohydrate content was obtained. After fermentation, a significant reduction (*P* < 0.05) in protein content for SD + S, SD + S + LAB8014, and W + S + LAB8014 broths was also observed (−18% on average).

**TABLE 4:** Biochemical composition of lyophilised broths at the start (0 h) and after 72 h of fermentation.

	Fermentation times (h)									
	
	0					72				
		
	Carbohydrate	Lipid	Protein	Ash	Moisture	Carbohydrate	Lipid	Protein	Ash	Moisture
Soybean drink	14.56 ± 1.02^*a*^	34.97 ± 1.52^*b*^	40.99 ± 3.49^*a*^	8.02 ± 1.03^*a*^	0.74 ± 1.05^*b*^	8.79 ± 2.02^*b*^	43.17 ± 1.31^*a*^	45.13 ± 3.36^*a*^	0.58 ± 1.32^*b*^	2.78 ± 1.73^*a*^
Soybean drink + LAB8014	13.37 ± 1.45^*a*^	35.15 ± 0.94^*b*^	40.97 ± 2.43^*b*^	11.13 ± 1.16^*a*^	0.54 ± 0.34^*b*^	7.71 ± 0.60^*b*^	44.33 ± 2.10^*a*^	45.25 ± 2.52^*a*^	1.05 ± 0.67^*b*^	4.44 ± 1.53^*a*^
Soybean drink + spirulina	15.29 ± 0.91^*a*^	20.00 ± 0.36^*b*^	60.95 ± 1.47^*a*^	0.24 ± 2.08^*b*^	2.96 ± 0.94^*b*^	9.66 ± 0.22^*b*^	24.88 ± 1.15^*a*^	43.35 ± 1.49^*b*^	8.89 ± 0.98^*a*^	11.47 ± 0.28^*a*^
Soybean drink + spirulina + LAB8014	16.31 ± 1.47^*a*^	14.91 ± 0.97^*b*^	57.82 ± 0.95^*a*^	6.27 ± 0.62^*a*^	4.75 ± 0.49^*b*^	12.23 ± 1.17^*b*^	23.40 ± 0.46^*a*^	51.32 ± 1.88^*b*^	5.74 ± 1.46^*a*^	7.66 ± 0.95^*a*^
Water + spirulina	16.05 ± 1.04^*a*^	9.05 ± 0.48^*b*^	67.42 ± 4.02^*a*^	3.18 ± 2.46^*b*^	3.08 ± 1.54^*a*^	14.48 ± 0.48^*b*^	10.98 ± 0.28^*a*^	64.83 ± 2.46^*a*^	7.61 ± 0.40^*a*^	4.22 ± 0.14^*a*^
Water + spirulina + LAB8014	16.29 ± 2.93^*a*^	7.73 ± 1.08^*b*^	63.62 ± 0.79^*a*^	1.64 ± 1.72^*b*^	10.22 ± 1.69^*a*^	12.77 ± 1.00^*b*^	10.31 ± 0.39^*a*^	54.83 ± 3.83^*b*^	8.92 ± 1.72^*a*^	11.48 ± 1.93^*a*^

*Data are expressed as % of broths powder. Values are expressed as mean ± s.d. Different letters in a row for the same biochemical component denote a significant difference (*P* < 0.05).*

At the end of fermentation, all the broths presented a significant (*P* < 0.05) increase in lipid content (+31% on average).

### *In vitro* Digestibility of Lyophilised Broths

Despite an average digestibility increase of +4% for SD, SD + LAB8014, SD + S, and SD + S + LAB8014 broths, fermentation did not significantly (*P* > 0.05) improve digestibility, except for W + S and W + S + LAB8014 broths (+7%, *P* < 0.05) (Table 5).

**TABLE 5:** *In vitro* digestibility (% dry matter) of lyophilised broths at the start (0 h) and after 72 h of fermentation.

	Fermentation times (h)	
	
	0	72
Soybean drink	74.58 ± 2.54^*a*^	78.54 ± 3.45^*a*^
Soybean drink + LAB8014	72.83 ± 3.78^*a*^	75.91 ± 2.16^*a*^
Soybean drink + spirulina	82.62 ± 2.02^*a*^	86.17 ± 1.85^*a*^
Soybean drink + spirulina + LAB8014	82.69 ± 1.42^*a*^	84.17 ± 1.38^*a*^
Water + spirulina	84.27 ± 1.41^*b*^	90.14 ± 1.75^*a*^
Water + spirulina + LAB8014	82.09 ± 0.09^*b*^	87.89 ± 1.66^*a*^

*Values are expressed as mean ± s.d. Different letters in a row denote a significant difference (*P* < 0.05).*

### Phycocyanin, Phenolic Content and Antioxidant Capacity of Lyophilised Broths

**FIGURE 4:**
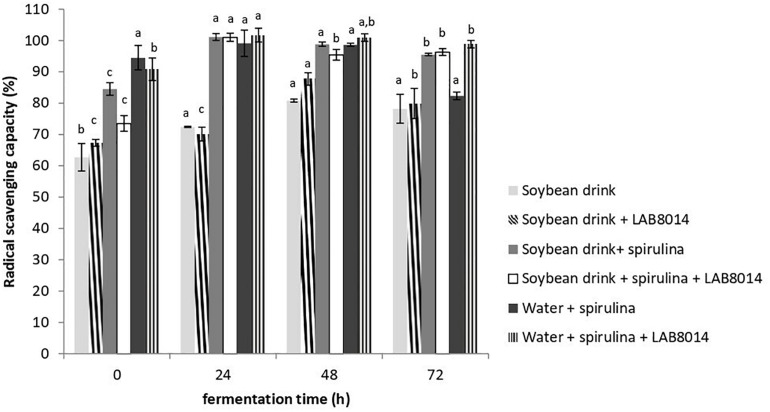
DPPH radical scavenging capacity (%) of lyophilised broths. Values are expressed as mean ± s.d. Different letters between columns of the same broth show significant differences (*P* < 0.05).

The 2,2-diphenyl-1-picrylhydrazyl (DPPH) radical scavenging capacity of lyophilised broths at the different fermentation times is shown in Figure 4. From the start (0 h) to one day of fermentation radical scavenging capacity increased (+16% on average). From 48 h to the end of fermentation (72 h) a general decrease in radical scavenging capacity in all the broths was observed (−6% on average). It is worth pointing out that after 72 h of fermentation all the broths still contain high DPPH radical scavenging capacity (>75%). At the end of fermentation, the SD + S + LAB8014 and W + S + LAB8014 broths showed the highest radical scavenging capacities compared to the other broths (96.3 and 98.8%, respectively).

**FIGURE 5:**
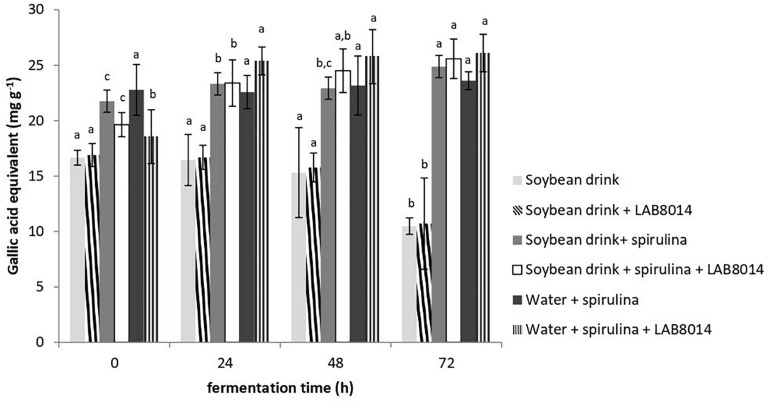
Total phenolic content, expressed as mg of gallic acid equivalent (GAE) per g of lyophilised broth. Values are expressed as mean ± s.d. Different letters between columns of the same broth show significant differences (*P* < 0.05).

In accordance with the DPPH results, at the end of fermentation the SD + S + LAB8014 and the W + S + LAB8014 broths showed the highest total phenolic content compared to the other broths (25.6 and 26.1 mg GAE g^–1^, respectively) (Figure 5). The broths with SD + S and W + S showed slightly lower phenolic content (about 25 mg GAE g^–1^). The lowest total phenolic content was found in SD and in SD + LAB8014 broths (about 11 mg GAE g^–1^).

**FIGURE 6:**
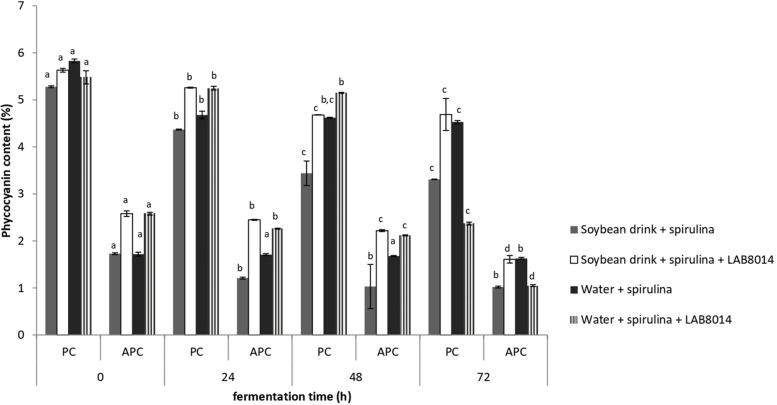
Phycocyanin contents of lyophilised fermentation broths containing *A. platensis* F&M-C256 biomass. Values are expressed as mean ± SD. Different letters between columns of the same broth, for PC or for APC, show significant differences (*P* < 0.05).

The phycocyanin contents of the different broths containing *A. platensis* F&M-C256 biomass is shown in Figure 6. During the entire fermentation process, PC was prevalent compared to the APC complex. After 72 h of fermentation, phycocyanin (PC + APC) decreased on average by −34%. Despite the general phycocyanin reduction, a high phycocyanin content was still present in the SD + S + LAB8014 and in W + S broths after fermentation (6.2% on average). Lower phycocyanin contents in SD + S and in W + S + LAB8014 broths were detected (4.3 and 3.4%, respectively).

### Intracellular Oxidation of Lyophilised Broths

**FIGURE 7:**
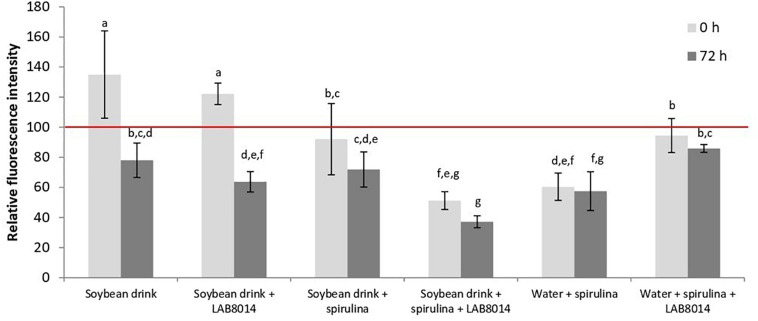
Intracellular oxidation in the yeast *S. cerevisiae* after 2-h exposure to different water extracts prepared from lyophilised broths at the start (0 h) and after 72 h of fermentation. The extracts were added to the yeast cell suspension (YCS) at a concentration of about 50 μL extract mL^– 1^ of YCS. The positive control (yeast cells treated with PBS buffer instead of extract) presents 100% relative fluorescence intensity (red line). Data are expressed as means of relative values (fluorescence intensity/optical density) ±s.d. Different letters between columns at particular time point show significant differences (*P* < 0.05).

The intracellular oxidation in *S. cerevisiae* after 2-h exposure to water extracts prepared from different lyophilised broths at the start (0 h) and after 72 h of fermentation is shown in Figure 7. Unfermented *A. platensis* F&M-C256 biomass extract was able to strongly reduce the intracellular oxidation level in yeast cells (−46%, result not shown) compared to the control (yeast cells treated with PBS buffer instead of extract). At the start of fermentation, only SD and SD + LAB8014 broths appear to have pro-oxidative behavior. At the end of fermentation (72 h), a decrease in intracellular oxidation was observed with all the broths.

After adding *A. platensis* F&M-C256 biomass to the broths, an average reduction of 25.5% in intracellular oxidation level compared to control was obtained. At the end of fermentation, *A. platensis* F&M-C256-based broths presented a further reduction in oxidative processes (−36.9% on average). The extract from SD + S + LAB8014 broth demonstrated to be the most powerful in reducing intracellular oxidation level, both at the start (−48.8%) and after 72 h of fermentation (−62.9%), compared to the other spirulina-based broths.

The intracellular oxidation results found for W + S broth at 0 h of fermentation (−40%), confirmed what has been found for the extract prepared from unfermented *A. platensis* F&M-C256 biomass. Unexpectedly, the extract prepared from W + S + LAB8014 broth showed a weak reduction in oxidative processes both at 0 h and after 72 h of fermentation (−5.5 and −14.1%, respectively).

**FIGURE 8:**
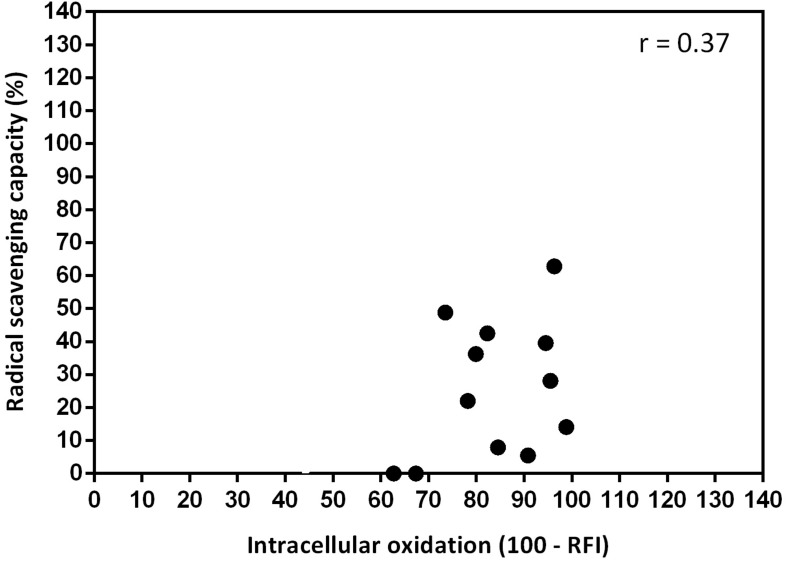
Linear correlation between *in vitro* radical scavenging capacity (expressed as %) and *in vivo* intracellular oxidation (expressed as 100 – relative fluorescence intensity, RFI) (*n* = 12; significance level *P* < 0.05). Negative values of RFI were considered as 0.

No significant (*P* > 0.05) correlation (*r* = 0.37) between *in vitro* radical scavenging capacity and *in vivo* intracellular oxidation was found (Figure 8).

## Discussion

Recently, because of the rising incidence of lactose intolerance, veganism, and health-conscious people, increased demand for fortified dairy-free food and beverage products with higher nutritional quality was observed (Di Cagno et al., 2013; de Marco Castro et al., 2019). Seaweed and microalgae represent appropriate substrates for the production of probiotic lactose-free foods and beverages through lactic acid fermentation due to their high nutritional value and bioactive components (Gupta et al., 2011; Niccolai et al., 2019a; Ścieszka and Klewicka, 2019). This study investigated the use of *A. platensis* F&M-C256 biomass in a vegetal soybean drink or in water, as substrate for lactic acid fermentation by the probiotic bacterium *L. plantarum* ATCC 8014, evaluating the fermented products in terms of bacteria content.

### Suitability of *A. platensis* F&M-C256 Biomass for *L. plantarum* ATCC 8014 Growth

As shown by bacterial growth curves (Figure 2), *A. platensis* F&M-C256, in water as well as in combination with the soybean drink, demonstrated to be an appropriate substrate for LAB8014 growth. To the best of our knowledge, only the recent work of Martelli et al. (2020b) focused on *A. platensis* fermentation in a non-dairy milk, specifically in a soybean drink. In accordance with the results obtained by Martelli et al. (2020b) we have demonstrated that *A. platensis* can promote LAB growth. Similar results have also been reported by Bhowmik et al. (2009), where the addition up to 10 g L^–1^ of *A. platensis* to MRS broth media containing different *Lactobacillus* cultures promoted the growth until 9 log CFU mL^–1^ (after 10 h), starting from a concentration of about 2.5 log CFU mL^–1^. Fermentation with four *Lactobacillus* strain (*L. plantarum* B7, *L. plantarum* C8-1, *Lactobacillus acidophilus* NCFM, and *L. plantarum* 121) and *Bacillus subtilis* improved deodorization of off-flavor and protein hydrolysis, also yielding an improved ratio of essential-to-total amino acids, compared to the unfermented spirulina and consequently, enhancing the sensory and antioxidant capacity during product development (Bao et al., 2018). Choi et al. (2018) also demonstrated an enhanced antioxidant capacity and β-carotene profile, upon *L. plantarum* HY-08 fermentation and ultrasonic extraction of *Arthrospira maxima*. The booster effects of *Arthrospira* sp. on the growth of LAB can be due to its high amount of nutritious substances such as free amino acids, exopolysaccharides, vitamins, and minerals, which stimulate bacterial metabolism (Parada et al., 1998; Beheshtipour et al., 2013). Several authors (Gardner et al., 2001; Bergqvist et al., 2005; Yoon et al., 2006; Limón et al., 2015; Nguyen et al., 2019; Ricci et al., 2019a; Thompson et al., 2020) tested the growth of LAB on vegetable matrixes, such as beet, carrot, onion, cabbage, dragon fruit, and pineapple-based juices, and bean extracts. Despite the bacterial initial concentration was always higher than in the present work, the maximum concentration reached by various species of *Lactobacillus* was similar to that found in our study varying between 9 and 10 log CFU mL^–1^ after 24 h in carrot juice and pineapple juice (Bergqvist et al., 2005; Nguyen et al., 2019), after 48 h in cabbage juice, elderberry juice, and bean extract (Yoon et al., 2006; Limón et al., 2015; Ricci et al., 2019a), and after 72 h in a vegetable mixture (Gardner et al., 2001).

To obtain a positive health effect from probiotics consumption, a level between 8 and 10 log CFU of live microorganisms per day for 1–2 weeks is required (Vanderhoof and Young, 1998). There are increasing evidences in favor of the claims of beneficial effects attributed to probiotics, including improvement of intestinal health, enhancement of the immune response, reduction of serum cholesterol, and cancer prevention (Markowiak and Śliżewska, 2017). Considering the high concentration of bacterial cells (from 8.8 to 10.7 log CFU mL^–1^) in the fermented spirulina-based broths reached in this study, we can assume that this cyanobacterium is a promising substrate for the obtainment of fermented beverages.

The pH trend and organic acids production during fermentation are significant parameters which is worth monitoring to evaluate the proper progress of fermentation. In the present work, at the start of fermentation, *A. platensis* F&M-C256-based broths showed a lower pH compared to the broths without spirulina biomass. The pH found for W + S broth at time 0 (6.1) is in accordance with the findings of de Marco Castro et al. (2019) a the beginning of fermentation. Further investigations aimed to clarify which are the *A. platensis* F&M-C256 compounds responsible for acidification are necessary. The decrease in pH, phenomenon indicating the correct progress of fermentation, is a result of the bacterial production of organic acids, primarily lactic and acetic acid (de Marco Castro et al., 2019). Moreover, in the food and beverage industry lactic and acetic acid are primarily used as a preservative, antibacterial, flavor enhancer, and acidulant (Sahasrabudhe and Sankpal, 2001). Knowing that *L. plantarum* has a facultative heterofermentative metabolism (Salvetti et al., 2012), it is plausible that in our study during the first 24 h of fermentation a high amount of lactic acid was produced and after 1 day, acetic acid became prevalent (Figure 3 and Table 3). Facultative heterofermentative species ferment hexoses to lactic acid via Embden-Meyerhof-Parnas pathway and are able to degrade pentoses and gluconate via an inducible phosphoketolase, an enzyme of the pentose phosphate pathway, with resulting production of acetic acid, under glucose limitation (Salvetti et al., 2012).

Fermentation of seaweeds and microalgae (such as *Chlorella*, *Tetraselmis*, and *Nannochloropsis*) led to a lactic acid production (1–5 g L^–1^) comparable to that found in the present study with all tested broths (Gupta et al., 2011; Gupta and Abu-Ghannam, 2012; Uchida and Miyoshi, 2013). Hwang et al. (2012) studied the lactic acid fermentation of a hydrolysate prepared from the seaweed *Ulva prolifera.* In accordance with the results obtained in the present study for SD + S and for SD + S + LAB8014 broths Hwang et al. (2012) found that after 24 h of incubation *Lactobacillus rhamnosus* was able to produce up to 4.3 g L^–1^ of lactic acid. In the study of Nguyen et al. (2012), the microalga *Hydrodictyon reticulum* was used as substrate for the production of lactic acid by *Lactobacillus paracasei* LA104. After 24 h of incubation, a higher lactic acid concentration (37 g L^–1^) compared to our study was reached. Also cereals have been studied as substrate for lactic acid fermentation. When coupled with enzymatic hydrolysis, *Lactobacillus delbrueckii* IFO 3202 was able to produce up to 28 g L^–1^ lactic acid from 100 g L^–1^ of rice bran (Tanaka et al., 2006). The greater yield of lactic acid obtained by some authors with different algae or food matrixes may be explained by faster sugars release times induced by enzymes addition and/or by the higher carbohydrate content and different carbohydrate profile. It is worth pointing out that rice bran contains 11% cellulose + hemicellulose and 47% starch + dextrin (Tanaka et al., 2006), while, *A. platensis* F&M-C256 biomass only contains 13% of total carbohydrates (Table 1).

### Nutritional Composition and *in vitro* Digestibility of Lyophilised Broths

A balanced nutritional composition, nutrient bioavailability, and digestibility are among the most important requirements to consider for the development of novel functional foods or beverages (Minekus et al., 2014).

The ability of LAB to hydrolyse and/or ferment proteins (Fang, 2002) may have significantly reduced the protein content for SD + S, SD + S + LAB8014, and W + S + LAB8014 broths after fermentation. Likely, also bacteria associated with *A. platensis* F&M-C256 biomass can hydrolize proteins; however, in the W + S broth they were not in sufficient amount to make this phenomenon appreciable. Tsakalidou et al. (1999) reported that, since the concentration of free amino acids and peptides present in milk is not sufficient for the growth of autochthonous LAB, their complex proteolytic system degrades mainly caseins into small peptides and amino acids, which fulfill their nutritional needs. However, the amount of protein still present in the SD + S + LAB8014 and in W + S + LAB8014 broths was >50% of the broth dry weight (Table 4). Protein degradation during food or beverage fermentation can lead to the production of volatile compounds (such as aromatic aldehydes or amines) conferring flavors, that can improve palatability and sensory properties of the final product (Urbach, 1997). Considering that in our work a reduction of proteins occurred at the end of fermentation, it is probable that bacteria broken down the proteins in peptides, which could have been later hydrolysed into amino acids by peptidases and then transformed in aromatic aldehydes or amines through transaminases and decarboxylases (Fang, 2002). The fermented broths could contain volatile compounds, able to confer aromatic properties (Fang, 2002). In order to identify the specifics volatile compounds produced by bacteria, HPLC and sensory analyses are necessary. It is also worth noting that the protein content in SD and SD + LAB8014 broths increased after fermentation, as if carbohydrates were turned into bacterial biomass that is richer in protein than soybean drink. This could also mean that the consumption of *A. platensis* F&M-C256 protein is higher than the −18% decrease observed (Table 4).

In the present work, at the end of fermentation all the broths presented a significant (*P* < 0.05) increase in lipid content. Several studies reported that the addition of LAB to dairy products may contribute to the production of free fatty acids by lipolysis of milk fat (Kurmann, 1988; Coşkun and Ondül, 2004; Yadav et al., 2007). Yadav et al. (2007) found that the addition of probiotic *L. acidophilus* and *Lactobacillus casei* to fermented milk resulted in higher lipolytic activity than milk without lactobacilli inoculation and produced more free fatty acids. Furthermore, *Lactobacillus* also have the ability to convert linoleic acid (LA) to conjugated linoleic acid (CLA). This attracted attention as a novel beneficial functional lipid, considering that CLAs have many positive health effects including reduced risk of carcinogenesis, atherosclerosis, obesity, improved hyperinsulinemia and prevention of catabolic effects of the immune system (Ogawa et al., 2005). In this study, LAB8014 could have produced fatty acids by using the lipid fraction of the soybean drink or of spirulina biomass, boosting the lipid content in the broths at the end of fermentation. Considering that *A. platensis* F&M-C256 biomass contains LA (1.15%) (Niccolai et al., 2019c) and that the most abundant fatty acid in soybean is LA (Sangwan et al., 1986), it is probable that during fermentation bacteria converted LA to CLA further improving the beneficial properties of the fermented broths. In the case of SD broth, without bacterial inoculation, the presence of LAB8014 after 48 h of fermentation could have lead to the same phenomena.

The *in vitro* digestibility provides useful information concerning the nutrient bioavailability of a specific product (Boisen and Fernández, 1997). Only few studies focus on the *in vitro* digestibility of microalgae (Mišurcovà et al., 2010; Tibbetts et al., 2012; Machů et al., 2014). In this study, fermentation did not improve digestibility, except for W + S and W + S + LAB8014 broths (Table 5). A similar increase of digestibility (+4.4%) was also reported by Niccolai et al. (2019a) for a broth containing water and *A. platensis* F&M-C256 biomass as the sole substrate for lactic acid fermentation by LAB8014. It is worth highlighting that at the end of fermentation, a large fraction of the solid residue, especially for SD + S + LAB8014 and for W + S + LAB8014 broths, was composed of LAB8014 cells, which have low digestibility (Alfano et al., 2015). The high presence of undigestible bacterial cells may explain the limited increase in digestibility after fermentation.

### Bioactive Potential of Lyophilised Broths: Phycocyanin, Phenolic Content and Antioxidant Capacity

Phycocyanin and polyphenols are the main bioactive components responsible for *Arthrospira* radical scavenging, antioxidant, and anti-inflammatory activities (Wu et al., 2016; Shabana et al., 2017). In the present work, at the end of fermentation the SD + S + LAB8014 and W + S + LAB8014 broths showed a high radical scavenging capacity (>95%) (Figure 4). Liu et al. (2011) reported a lower radical scavenging capacity (<35%) for *A. platensis* biomass fermented 48 h in cow milk. In a recent work (de Marco Castro et al., 2019) aimed at improving the bioactive profile of *A. platensis* F&M-C256 biomass through fermentation with LAB8014, after 24 h of fermentation the DPPH radical scavenging capacity increased up to 60%, finding a lower increase compared to that obtained in the present study for spirulina-based broths.

Phenolic compounds, recognized as strong antioxidants, are able to scavenge ROS (Cai et al., 2004). *Arthrospira* spp. can contain different quantities of phenolics (generally from 5 to about 50 mg GAE g^–1^), depending on strain characteristics and culture conditions (Abd El-Baki et al., 2009; Kepekçi and Saygideger, 2012). In a study by Liu et al. (2011), after 48 h, fermented milk with *A. platensis* biomass contained 33.6 mg GAE g^–1^ and it was hypothesized that lactic acid fermentation helps to release polyphenols from spirulina with improved biological activity.

Similar to the results obtained in this work (Figure 5), Niccolai et al. (2019a) reported that the total phenolic content of a broth containing water + spirulina + LAB8014 increased from the start, up to the second day of fermentation. In the study of de Marco Castro et al. (2019), *A. platensis* biomass fermented by LAB8014 for 48 h led to a total phenolic content of 15 mg GAE g^–1^, which is lower to that found in this work for spirulina-based broths after two days of fermentation (24 mg GAE g^–1^ on average).

In our work, the results of DPPH radical scavenging capacity and of total phenolic content are nearly superimposable. Therefore, phenolics are one of the main responsible for the antioxidant activity, probably together with phycocyanin released from *A. platensis* F&M-C256 biomass and with compounds that have antioxidant potential from soybean drink and from LAB8014. In accordance with the positive significant (*P* < 0.05) correlation between total phenolic content and DPPH radical scavenging capacity (*r* = 0.85, results not shown) for the broths fermented 72 h found in this study, several authors (Meda et al., 2005; Paixao et al., 2007; Fu et al., 2011) also reported significant positive correlations between total phenolics and antioxidant activity for different foods or beverages. Many studies also show that *L. plantarum* strains present antioxidant activity (Aguilar-Toalá et al., 2017; Wang et al., 2017; Min et al., 2019). Suzuki et al. (2013) identified L-3-(4-hydroxyphenyl) and L-indole-3-lactic acid as possible responsible for DPPH radical scavenging capacity of *L. plantarum* cultures. After 72 h, the fermented broths contained a high concentration of LAB8014, representing another factor (in addition to phenolics and phycocyanin from *Arthrospira*) which may have entailed the increase in radical scavenging capacity.

Phycocyanin is a well known water-soluble pigment-protein complex from the light-harvesting phycobiliprotein family contained in cyanobacteria (Eriksen, 2008). Phycocyanins include two principal pigment complexes, allophycocyanin (APC) and phycocyanin (PC) that are in turn constituted by subunits with a different structure which present specific peaks of fluorescence emission (660 and 646 nm, respectively) or absorption (650 and 617 nm, respectively) in the visible region (Glazer and Hixson, 1975; Chapman et al., 1967). Phycocyanin has been used as a natural dye for food, cosmetics, and in the pharmaceutical field (Eriksen, 2008). Phycocyanin demonstrated great pharmaceutical potential in *in vitro* and *in vivo* trials due to its anti-inflammatory activity (it represses nitric oxide synthase expression, reduces nitrite synthesis, and prevents glucose oxidase-induced edema) (Romay et al., 1998; Cherng et al., 2007), prevention of cholesterol-induced atherosclerosis (Riss et al., 2007), anti-platelet aggregation effect (inhibition of cyclooxygenase) (Chiu et al., 2006), and anti-carcinogenic action (it induces apoptosis and inhibits proliferation of human myeloid leukemia cells) (Roy et al., 2007). *A. platensis* is the main source of phycocyanin (Wu et al., 2016). In our study, after 72 h of fermentation phycocyanin decreased for all the broths (−34% on average) (Figure 6). These results are in accordance with those reported by de Marco Castro et al. (2019), who found a reduction of phycocyanin content in a broth containing *A. platensis* F&M-C256 biomass after 72 h of fermentation with LAB8014. Phycocyanin is a temperature-sensitive pigment (Sarada et al., 1999), which, under the fermentation conditions set for this work (37°C), might have been degraded after 72 h. Moreover, it is probable that LAB to satisfy the nitrogen nutritional requirement consumed phycocyanin, that represents 8–10% of spirulina proteins (Wu et al., 2016), for their metabolism (Hebert et al., 2000; de Marco Castro et al., 2019), reducing the total phycocyanin content in the broths during fermentation. Liu et al. (2011) hypothesized that LAB are able to use phycocyanin during fermentation converting it into phycocyanobilin, probably through proteolysis (Savijoki et al., 2006).

Bhat and Madyastha (2000) demonstrated that phycocyanin efficiently scavenges peroxynitrite (of about 90%), a potent physiological inorganic toxin, showing that the pigment significantly inhibits the peroxynitrite-mediated single-strand breaks in supercoiled plasmid DNA. In the present study, the spirulina-based broths after fermentation still contained a high phycocyanin concentration, then it is possible to conclude that the presence of the pigment, able to scavenge oxygen-free radicals, besides inorganic toxins, contributed (together with phenolics) to the high radical scavenging activity observed.

Besides the *in vitro* measuring of radical scavenging the intracellular antioxidant capacity assay was developed to accurately measure the antioxidant potential of dietary supplements, foods, beverages or of specific antioxidants in the cells (Wolfe and Liu, 2007). Considering that the nature and role of enzymes participating in decreasing ROS level, repair damaged macromolecules and eliminate irreparable proteins are similar at all levels of cellular organization, yeasts are useful model organisms for studying the various aspects of oxidative stress at the biochemical, molecular and cellular level (Sigler et al., 1999). The yeast *S. cerevisiae* is an appropriate model organism to evaluate eukaryotic cellular processes involved in the antioxidative activity following treatment with bioactive compounds (Cigut et al., 2011; Zakrajsek et al., 2011). The soybean drink is a nutrient-rich substrate for *S. cerevisiae*, therefore, yeasts can immediately start increasing their metabolic activity using these nutrients. As a result of the increased metabolic activity, increased oxidation level in yeasts is expected (Scharf et al., 1998). This may explain the pro-oxidative behavior observed in our study for soybean drink and soybean drink + LAB8014 broths (Figure 7). Despite some beneficial health properties related to soybean consumption (Devine, 2002), several studies demonstrated that soy-based foods can contain anti-nutritional factors such as protease inhibitors, hemagglutinating isolectins, and phytates (Bajpai et al., 2005), that may have played some role in oxidative processes.

After fermentation soybean drink nutrients may have been completely consumed by bacteria, reasonably in the first 48 h of fermentation (see the case of bacterial growth in the SD + LAB8014 broth). During fermentation *Lactobacillus* can produce bioactive peptides from soybean proteins with antioxidative activity (Ewe et al., 2011). In the present study, in addition to the antioxidant action from *A. platensis* F&M-C256, bioactive peptides could have interacted with isoflavone aglycones contained in the fermented soybean drink, that present well known antioxidative activity (Di Cagno et al., 2010), probably causing the reduction of oxidative processes. LAB have capabilities of converting isoflavone glucosides to free aglycones with a higher number of hydroxyl groups or lower steric hindrance to hydroxyl groups, further enhancing the antioxidant activity (Chun et al., 2007; Zhao and Shah, 2016). It is also possible that after 72 h of fermentation, bacteria started to lyse releasing their intracellular components that could contribute to the oxidative processes reduction in the yeast cells (Aruoma and Halliwell, 1987; Miller and Britigan, 1997). The presence of other antioxidants, that can be contained in fermented food products, such as milk (e.g., antioxidant enzymes, CLA, coenzyme Q_10_, lactoferrin, vitamins C, E, A, and D_3_, equol, uric acid, carotenoids, and mineral activators of antioxidant enzymes) (Fardet and Rock, 2018), could explain the reduction in intracellular oxidation after 72 h of fermentation obtained also for the broth containing only soybean drink.

At the end of fermentation, *A. platensis* F&M-C256-based broths present a further reduction in oxidative processes, maybe due to the release of antioxidant components contained in *A. platensis* F&M-C256 (mainly phycocyanin and phenolics). The powerful effect in reducing intracellular oxidation level demonstrated by SD + S + LAB8014 broth could be related to phenolics and phycocyanin from cyanobacterial biomass that are released during fermentation and may have acted in a synergistic effect with LAB8014 and their products and isoflavone aglycones causing the strong reduction in intracellular oxidation. The weak reduction in intracellular oxidation showed by the extract prepared from W + S + LAB8014 broth suggests that lactic acid fermentation of spirulina as a sole substrate is not so efficient in antioxidative activity, as in the case, where SD + S + LAB8014 broth was fermented. Further investigations are necessary to fully clarify this point.

In general, our data show that the sensitivity of the *in vivo* intracellular oxidation model, a method based on a vital microorganism (*S. cerevisiae*), is higher compared to that of the *in vitro* model. In accordance with these results, several authors (Slatnar et al., 2012; Amaretti et al., 2013; Fardet and Rock, 2018) showed no significant correlation between *in vitro* and *in vivo* antioxidant trials.

Despite many works dealt with *A. platensis* biomass potential health benefits (Colla et al., 2008; Kim et al., 2010; Gutiérrez-Rebolledo et al., 2015; Bigagli et al., 2017), to the best of our knowledge, no literature is available concerning the inhibition of intracellular oxidation of microalgae-based beverages fermented with LAB. In a study of Slatnar et al. (2012) where the main objective was to determine the *in vitro* and *in vivo* antioxidant activity of different berry juices, a similar reduction in intracellular oxidation for blueberry and bilberry juices (about −40%) compared to the SD + LAB8014, SD + S, and W + S broths (fermented 72 h) of our work was found. Considering that *A. platensis* F&M-C256-based broths showed similar or higher antioxidant potential compared to blueberry and bilberry juices, that are recognized as powerful antioxidant sources between vegetables (Slatnar et al., 2012), it is possible to understand the health potential for the consumers. The new lactose-free beverages obtained in this study (especially the SD + S + LAB8014 beverage), could be included in the daily diet, entering the food/beverage market, making these functional products attractive for investors of the algae sector, which in 2018 reached USD 3.98 billion and it is expected it will grow at a CAGR of 5.4% in the period 2018–2023 (Markets and Markets Report, 2018).

## Conclusion

In conclusion, *A. platensis* F&M-C256 biomass demonstrated to be a suitable substrate for *L. plantarum* ATCC 8014 growth. At the end of the fermentation, LAB8014 cells in SD + S + LAB8014 and W + S + LAB8014 broths constituted about 25 and 17% of the broth dry weight. The amount of protein still present was more than 50% of the broths dry weight. Total phenolic content, *in vitro* and *in vivo* antioxidant activity increased, while phycocyanin content decreased. A significant improvement in digestibility for W + S + LAB8014 broth was observed after fermentation, while no significant increase for SD + S + LAB8014 broth was found.

Fermentation by LAB8014 is an appropriate technology to obtain *A. platensis-*based lactose-free beverages. Future steps to reach the full development of the functional beverages in order to get to the market will be the determination of the technological properties (stability of components, safety, etc.) and sensorial aspects. The new functional beverages could be daily used by vegan, vegetarian, sportsman, children, elderlies, and health-conscious people making these functional products attractive for companies that intend to invest in nutraceuticals and/or in the algae sector.

## Data Availability Statement

The raw data supporting the conclusions of this article will be made available by the authors, without undue reservation.

## Author Contributions

AN, PJ, and MT oversaw the conception and design. AN contributed to the fermentation trials, microbiological analyses, biochemical composition, phycocyanin, phenolics, *in vitro* antioxidant capacity determination, and drafted the manuscript and statistical analyses. EZ contributed to the organic acids determination. AN, KB, and PJ contributed to *in vitro* digestibility test and *in vivo* intracellular oxidation determination. AN, KB, LR, NB, MT, and PJ participated in data analysis, discussion of the results, and revision of the manuscript. All authors contributed to the article and approved the submitted version.

## Conflict of Interest

*Arthrospira platensis* F&M-C256 belongs to the Microalgae Culture Collection of Fotosintetica & Microbiologica S.r.l., in which MT and LR have a financial interest. LR and MT are employed at the University of Florence. LR is shareholder and the President of the Board of Fotosintetica & Microbiologica S.r.l. The remaining authors declare that the research was conducted in the absence of any commercial or financial relationships that could be construed as a potential conflict of interest.

## References

[B1] Abd El-Baki H. H. El Baz F. K. El-Baroty G. S. (2009). Production of phenolic compounds from *Spirulina maxima* microalgae and its protective effects. *Afr. J. Biotechnol.* 8 7059–7067.

[B2] Abdulqader G. Barsanti L. Tredici M. R. (2000). Harvest of *Arthrospira platensis* from Lake Kossorom (Chad) and its household usage among the Kanembu. *J. Appl. Phycol.* 12 493–498. 10.1023/A:1008177925799

[B3] Abiusi F. Sampietro G. Marturano G. Biondi N. Rodolfi L. D’Ottavio M. (2014). Growth, photosynthetic efficiency, and biochemical composition of *Tetraselmis suecica* F&M-M33 grown with LEDs of different colors. *Biotechnol. Bioeng.* 111 956–964. 10.1002/bit.25014 23904253

[B4] Aguilar-Toalá J. E. Santiago-López L. Peres C. M. Peres C. Garcia H. S. Vallejo-Cordoba B. (2017). Assessment of multifunctional activity of bioactive peptides derived from fermented milk by specific *Lactobacillus plantarum* strains. *J. Dairy Sci.* 100 65–75. 10.3168/jds.2016-11846 27865495

[B5] Alfano A. Donnarumma G. Cimini D. Fusco A. Marzaioli I. De Rosa M. (2015). *Lactobacillus plantarum*: microfiltration experiments for the production of probiotic biomass to be used in food and nutraceutical preparations. *Biotechnol. Prog.* 31 325–333. 10.1002/btpr.2037 25582766

[B6] Amaretti A. Di Nunzio M. Pompei A. Raimondi S. Rossi M. Bordoni A. (2013). Antioxidant properties of potentially probiotic bacteria: in vitro and in vivo activities. *Appl. Microbiol. Biotechnol.* 97 809–817. 10.1007/s00253-012-4241-7 22790540

[B7] Aruoma O. I. Halliwell B. (1987). Action of hypochlorous acid on the antioxidant protective enzymes superoxide dismutase, catalase and glutathione peroxidase. *Biochem. J.* 248 973–976. 10.1042/bj2480973 2829848 PMC1148647

[B8] Bajpai S. Sharma A. Gupta M. N. (2005). Removal and recovery of antinutritional factors from soybean flour. *Food Chem.* 89 497–501. 10.1016/j.foodchem.2004.02.055

[B9] Bao J. Zhang X. Zheng J. H. Ren D. F. Lu J. (2018). Mixed fermentation of *Spirulina platensis* with *Lactobacillus plantarum* and *Bacillus subtilis* by random-centroid optimization. *Food Chem.* 264 64–72. 10.1016/j.foodchem.2018.05.027 29853406

[B10] Batista A. P. Niccolai A. Bursic I. Sousa I. Raymundo A. Rodolfi L. (2019). Microalgae as functional ingredients in savory food products: application to wheat crackers. *Foods* 8 1–22. 10.3390/foods8120611 31771197 PMC6963871

[B11] Batista A. P. Niccolai A. Fradinho P. Fragoso S. Bursic I. Rodolfi L. (2017). Microalgae biomass as an alternative ingredient in cookies: sensory. physical and chemical properties. antioxidant activity and in vitro digestibility. *Algal Res.* 26 161–171. 10.1016/j.algal.2017.07.017

[B12] Beheshtipour H. Mortazavian A. M. Mohammadi R. Sohrabvandi S. Khosrav-Darani K. (2013). Supplementation of *Spirulina platensis* and *Chlorella vulgaris* algae into probiotic fermented milks. *Compr. Rev. Food Sci. Food Saf.* 12 144–154. 10.1111/1541-4337.12004

[B13] Bergqvist S. W. Sandberg A. S. Carlsson N. G. Andlid T. (2005). Improved iron solubility in carrot juice fermented by homo-and hetero-fermentative lactic acid bacteria. *Food Microbiol.* 22 53–61. 10.1016/j.fm.2004.04.006

[B14] Bhat V. B. Madyastha K. M. (2000). C-Phycocyanin: a potent peroxyl radical scavenger *in vivo* and *in vitro*. *Biochem. Biophys. Res. Commun.* 275 20–25. 10.1006/bbrc.2000.3270 10944434

[B15] Bhowmik D. Dubey J. Mehra S. (2009). Probiotic efficiency of *Spirulina platensis*-stimulating growth of lactic acid bacteria. *World J. Dairy Food Sci.* 4 160–163.

[B16] Bigagli E. Cinci L. Niccolai A. Tredici M. R. Biondi N. Rodolfi L. (2017). Safety evaluations and lipid-lowering activity of an *Arthrospira platensis* enriched diet: a 1-month study in rats. *Food Res. Int.* 102 380–386. 10.1016/j.foodres.2017.09.011 29195962

[B17] Boisen S. Fernández J. A. (1997). Prediction of the total tract digestibility of energy in feedstuffs and pig diets by in vitro analyses. *Anim. Feed Sci. Technol.* 68 277–286. 10.1016/S0377-8401(97)00058-8

[B18] Borowitzka M. A. (2013). High-value products from microalgae-their development and commercialisation. *J. Appl. Phycol.* 25 743–756. 10.1007/s10811-013-9983-9

[B19] Cai Y. Luo Q. Sun M. Corke H. (2004). Antioxidant activity and phenolic compounds of 112 traditional Chinese medicinal plants associated with anticancer. *Life Sci.* 74 2157–2184. 10.1016/j.lfs.2003.09.047 14969719 PMC7126989

[B20] Chapman D. J. Cole W. J. Siegelman H. W. (1967). Chromophores of allophycocyanin and R-phycocyanin. *Biochem. J.* 105 903–905. 10.1042/bj1050903 16742564 PMC1198406

[B21] Cherng S.-C. Cheng S.-N. Tarn A. Chou T.-C. (2007). Anti-inflammatory activity of c-phycocyanin in lipopolysaccharide-stimulated RAW 264.7 macrophages. *Life Sci.* 81 1431–1435. 10.1016/j.lfs.2007.09.009 17961604

[B22] Chiu H.-F. Yang S.-P. Kuo Y.-L. Lai Y.-S. Chou T.-C. (2006). Mechanisms involved in the antiplatelet effect of C-phycocyanin. *Br. J. Nutr.* 95 435–440. 10.1079/BJN20051643 16469164

[B23] Choi W. Kang D. Heo S. J. Lee H. (2018). Enhancement of the neuroprotective effect of fermented *Spirulina maxima* associated with antioxidant activities by ultrasonic extraction. *Appl. Sci.* 8 1–12. 10.3390/app8122469

[B24] Christaki E. Florou-Paneri P. Bonos E. (2011). Microalgae: a novel ingredient in nutrition. *Int. J. Food Sci. Nutr.* 62 794–799. 10.3109/09637486.2011.582460 21574818

[B25] Chun J. Kim G. M. Lee K. W. Choi I. D. Kwon G. H. Park J. Y. (2007). Conversion of isoflavone glucosides to aglycones in soymilk by fermentation with lactic acid bacteria. *J. Food Sci.* 72 M39–M44. 10.1111/j.1750-3841.2007.00276.x 17995840

[B26] Cigut T. Polak T. Gasperlin L. Raspor P. Jamnik P. (2011). Antioxidative activity of propolis extract in yeast cells. *J. Agric. Food. Chem.* 59 11449–11455. 10.1021/jf2022258 21958212

[B27] Colla L. M. Muccillo-Baisch A. L. Costa J. A. V. (2008). *Spirulina platensis* effects on the levels of total cholesterol, HDL and triglycerides in rabbits fed with a hypercholesterolemic diet. *Braz. Arch. Biol. Techn.* 51 405–411. 10.1590/S1516-89132008000200022

[B28] Coşkun H. Ondül E. (2004). Free fatty acid accumulation by mesophilic lactic acid bacteria in cold stored milk. *J. Microbiol.* 42 133–138. 15357307

[B29] de Caire G. Z. Parada J. L. Zaccaro M. C. Storni de Cano M. M. (2000). Effect of *Spirulina platensis* biomass on the growth of lactic acid bacteria in milk. *World J. Microbiol. Biotechnol.* 16 563–565. 10.1023/A:1008928930174

[B30] de Jesus Raposo M. de Morais A. de Morais R. (2016). Emergent sources of prebiotics: seaweeds and microalgae. *Mar. Drugs* 14 1–27. 10.3390/md14020027 26828501 PMC4771980

[B31] de Marco Castro E. Shannon E. Abu-Ghannam N. (2019). Effect of Fermentation on Enhancing the Nutraceutical Properties of *Arthrospira platensis* (Spirulina). *Fermentation* 5 1–16. 10.3390/fermentation5010028

[B32] de Vries M. C. Siezen R. J. Wijman J. G. Zhao Y. Kleerebezem M. De Vos W. M. (2006). Comparative and functional analysis of the rRNA-operons and their tRNA gene complement in different lactic acid bacteria. *Systemat. Appl. Microbiol.* 29 358–367. 10.1016/j.syapm.2005.11.010 16338113

[B33] Devine D. (2002). Soya and health 2002–clinical evidence, dietetic applications. *Nutr. Bull.* 27 195–198. 10.1046/j.1467-3010.2002.00259.x

[B34] Di Cagno R. Coda R. De Angelis M. Gobbetti M. (2013). Exploitation of vegetables and fruits through lactic acid fermentation. *Food Microbiol.* 33 1–10. 10.1016/j.fm.2012.09.003 23122495

[B35] Di Cagno R. Mazzacane F. Rizzello C. G. Vincentini O. Silano M. Giuliani G. (2010). Synthesis of isoflavone aglycones and equol in soy milks fermented by food-related lactic acid bacteria and their effect on human intestinal Caco-2 cells. *J. Agr. Food Chem.* 58 10338–10346. 10.1021/jf101513r 20822177

[B36] Dubois M. Gilles K. A. Hamilton J. K. Rebers P. T. Smith F. (1956). Colorimetric method for determination of sugars and related substances. *Anal. Chem.* 28 350–356. 10.1021/ac60111a017

[B37] Eriksen N. T. (2008). Production of phycocyanin-a pigment with applications in biology, biotechnology, foods and medicine. *Appl. Microbiol. Biotechnol.* 80 1–14. 10.1007/s00253-008-1542-y 18563408

[B38] Ewe J. A. Wan-Abdullah W. N. Alias A. K. Bhat R. Liong M. T. (2011). ACE inhibitory activity and bioconversion of isoflavones by *Lactobacillus* in soymilk supplemented with B-vitamins. *Br. Food J.* 113 1127–1146. 10.1108/00070701111174578

[B39] Fang G. (2002). *Production and Utilization of Peptides in Lactococcus lactis.* Groningen: University Library Groningen.

[B40] Fardet A. Rock E. (2018). *In vitro* and *in vivo* antioxidant potential of milks, yoghurts, fermented milks and cheeses: a narrative review of evidence. *Nutr. Res. Rev*. 31 52–70. 10.1017/S0954422417000191 28965518

[B41] Fradinho P. Niccolai A. Soares R. Rodolfi L. Biondi N. Tredici M. R. (2020). Effect of *Arthrospira platensis* (spirulina) incorporation on the rheological and bioactive properties of gluten-free fresh pasta. *Algal Res.* 45:101743 10.1016/j.algal.2019.101743

[B42] Frirdich E. Gaynor E. C. (2013). Peptidoglycan hydrolases, bacterial shape, and pathogenesis. *Curr. Op. Microbiol.* 16 767–778. 10.1016/j.mib.2013.09.005 24121030

[B43] Fu L. Xu B. T. Xu X. R. Gan R. Y. Zhang Y. Xia E. Q. (2011). Antioxidant capacities and total phenolic contents of 62 fruits. *Food Chem.* 129 345–350. 10.1016/j.foodchem.2011.04.079 30634236

[B44] Ganesan P. Kumar C. S. Bhaskar N. (2008). Antioxidant properties of methanol extract and its solvent fractions obtained from selected Indian red seaweeds. *Biores. Technol.* 99 2717–2723. 10.1016/j.biortech.2007.07.005 17706415

[B45] Gardner N. J. Savard T. Obermeier P. Caldwell G. Champagne C. P. (2001). Selection and characterization of mixed starter cultures for lactic acid fermentation of carrot, cabbage, beet and onion vegetable mixtures. *Int. J. Food Microbiol.* 64 261–275. 10.1016/S0168-1605(00)00461-X 11294348

[B46] Glazer A. N. Hixson C. S. (1975). Characterization of R-phycocyanin. Chromophore content of R-phycocyanin and C-phycoerythrin. *J. Biol. Chem.* 250 5487–5495. 806593

[B47] Guldas M. Irkin R. (2010). Influence of *Spirulina platensis* powder on the microflora of yoghurt and acidophilus milk. *Mljekarstvo* 60 237–243.

[B48] Gupta S. Abu-Ghannam N. Scannell A. G. (2011). Growth and kinetics of *Lactobacillus plantarum* in the fermentation of edible Irish brown seaweeds. *Food Bioprod. Process.* 89 346–355. 10.1016/j.fbp.2010.10.001

[B49] Gupta S. Abu-Ghannam N. (2012). Probiotic fermentation of plant based products: possibilities and opportunities. *Crit. Rev. Food Sci. Nutr.* 52 183–199. 10.1080/10408398.2010.499779 22059963

[B50] Gutiérrez-Rebolledo G. A. Galar-Martínez M. García-Rodríguez R. V. Chamorro-Cevallos G. A. Hernández-Reyes A. G. Martínez-Galero E. (2015). Antioxidant effect of *Spirulina* (*Arthrospira*) *maxima* on chronic inflammation induced by Freund’s complete adjuvant in rats. *J. Med. Food.* 18 865–871. 10.1089/jmf.2014.0117 25599112 PMC4523079

[B51] Hebert E. M. Raya R. R. De Giori G. S. (2000). Nutritional requirements and nitrogen-dependent regulation of proteinase activity of *Lactobacillus helveticus* CRL 1062. *Appl. Environ. Microbiol.* 66 5316–5321. 10.1128/AEM.66.12.5316-5321.2000 11097908 PMC92462

[B52] Herrera A. Boussiba S. Napoleone V. Hohlberg A. (1989). Recovery of c-phycocyanin from the cyanobacterium *Spirulina maxima*. *J. Appl. Phycol.* 1 325–331. 10.1007/BF00003469

[B53] Hwang H. J. Kim S. M. Chang J. H. Lee S. B. (2012). Lactic acid production from seaweed hydrolysate of *Enteromorpha prolifera* (Chlorophyta). *J. Appl. Phycol.* 24 935–940. 10.1007/s10811-011-9714-z

[B54] Jakubowski W. Bartosz G. (1997). Estimation of oxidative stress in *Saccharomyces cerevisae* with fluorescent probes. *Int. J. Biochem. Cell Biol.* 29 1297–1301. 10.1016/S1357-2725(97)00056-3 9451827

[B55] Jiménez-Escrig A. Jiménez-Jiménez I. Pulido R. Saura-Calixto F. (2001). Antioxidant activity of fresh and processed edible seaweeds. *J. Sci. Food Agr.* 81 530–534. 10.1002/jsfa.842

[B56] Karczewski J. Troost F. J. Konings I. Dekker J. Kleerebezem M. Brummer R. J. M. (2010). Regulation of human epithelial tight junction proteins by *Lactobacillus plantarum* in vivo and protective effects on the epithelial barrier. *Am. J. Physiol.-Gastr. Liver Physiol.* 298 G851–G859. 10.1152/ajpgi.00327.2009 20224007

[B57] Kepekçi R. A. Saygideger S. D. (2012). Enhancement of phenolic compound production in *Spirulina platensis* by two-step batch mode cultivation. *J. Appl. Phycol.* 24 897–905. 10.1007/s10811-011-9710-3

[B58] Kim M. Y. Cheong S. H. Lee J. H. Kim M. J. Sok D. E. Kim M. R. (2010). Spirulina improves antioxidant status by reducing oxidative stress in rabbits fed a high-cholesterol diet. *J. Med. Food* 13 420–426. 10.1089/jmf.2009.1215 20210608

[B59] Kulshreshtha A. Jarouliya U. Bhadauriya P. Prasad G. B. K. S. Bisen P. S. (2008). Spirulina in health care management. *Curr. Pharm. Biotech.* 9 400–405. 10.2174/138920108785915111 18855693

[B60] Kurmann J. A. (1988). Starters of fermented milks: starters with selectedintestinal bacteria. *Int. Dairy Fed. Bull*. 227 41–55.

[B61] Limón R. Peñas E. Torino M. Martínez-Villaluenga C. Dueñas M. Frias J. (2015). Fermentation enhances the content of bioactive compounds in kidney bean extracts. *Food Chem.* 172 343–352. 10.1016/j.foodchem.2014.09.084 25442563

[B62] Liu J. G. Hou C. W. Lee S. Y. Chuang Y. Lin C. C. (2011). Antioxidant effects and UVB protective activity of *Spirulina* (*Arthrospira platensis*) products fermented with lactic acid bacteria. *Proc. Biochem.* 46 1405–1410. 10.1016/j.procbio.2011.03.010

[B63] Lowry O. H. Rosebrough N. J. Farr A. L. Randall R. J. (1951). Protein measurement with the Folin phenol reagent. *J. Biol. Chem.* 193 265–275. 14907713

[B64] Machů L. Mišurcová L. Samek D. Hrabě J. Fišera M. (2014). In vitro digestibility of different commercial edible algae products. *J. Aquat. Food Prod. Technol.* 23 423–435. 10.1080/10498850.2012.721873

[B65] Markets and Markets Report (2018). *Algae Products Market by Type, Application, Source, Form, and Region—Global Forecast to Report ID: 4521311. 2018.* Available online: https://www.researchandmarkets.com/publication/me4to4g/ (accessed January 10, 2020).

[B66] Markowiak P. Śliżewska K. (2017). Effects of probiotics, prebiotics, and synbiotics on human health. *Nutrients* 9:1021. 10.3390/nu9091021 28914794 PMC5622781

[B67] Marsh J. B. Weinstein D. B. (1966). Simple charring method for determination of lipids. *J. Lipid Res.* 7 574–576. 5965305

[B68] Martelli F. Alinovi M. Bernini V. Gatti M. Bancalari E. (2020b). *Arthrospira platensis* as natural fermentation booster for milk and soy fermented beverages. *Foods* 9 1–15. 10.3390/foods9030350 32197327 PMC7142653

[B69] Martelli F. Favari C. Mena P. Guazzetti S. Ricci A. Del Rio D. (2020a). Antimicrobial and fermentation potential of *Himanthalia elongata* in food applications. *Microorganisms* 8 1–15. 10.3390/microorganisms8020248 32069955 PMC7074776

[B70] Mazinani S. Fadaei V. Khosravi-Darani K. (2016). Impact of *Spirulina platensis* on physicochemical properties and viability of *Lactobacillus acidophilus* of probiotic UF feta cheese. *J. Food Proc. Pres.* 40 1318–1324. 10.1111/jfpp.12717

[B71] Meda A. Lamien C. E. Romito M. Millogo J. Nacoulma O. G. (2005). Determination of the total phenolic, flavonoid and proline contents in Burkina Fasan honey, as well as their radical scavenging activity. *Food Chem.* 91 571–577. 10.1016/j.foodchem.2004.10.006

[B72] Miller R. A. Britigan B. E. (1997). Role of oxidants in microbial pathophysiology. *Clin. Microbiol. Rev.* 10 1–18. 10.1128/CMR.10.1.1 8993856 PMC172912

[B73] Min W. H. Fang X. B. Wu T. Fang L. Liu C. L. Wang J. (2019). Characterization and antioxidant activity of an acidic exopolysaccharide from *Lactobacillus plantarum* JLAU103. *J. Biosci. Bioeng.* 127 758–766. 10.1016/j.jbiosc.2018.12.004 30600152

[B74] Minekus M. Alminger M. Alvito P. Ballance S. Bohn T. Bourlieu C. (2014). A standardised static in vitro digestion method suitable for food – an international consensus. *Food Funct.* 5 1113–1124. 10.1039/C3FO60702J 24803111

[B75] Mišurcovà L. Kráèmar S. Boøivoj K. Vacek J. (2010). Nitrogen content, dietary fiber, and digestibility in algal food products. *Czech J. Food Sci.* 28 27–35. 10.17221/111/2009-CJFS

[B76] Muhialdin B. J. Kadum H. Zarei M. Hussin A. S. M. (2020). Effects of metabolite changes during lacto-fermentation on the biological activity and consumer acceptability for dragon fruit juice. *LWT* 121:108992 10.1016/j.lwt.2019.108992

[B77] Nguyen B. T. Bujna E. Fekete N. Tran A. Rezessy-Szabo J. M. Prasad R. (2019). Probiotic beverage from pineapple juice fermented with *Lactobacillus* and *Bifidobacterium* strains. *Front. Nutr.* 6:54. 10.3389/fnut.2019.00054 31143765 PMC6521632

[B78] Nguyen C. M. Kim J.-S. Hwang H. J. Park M. S. Choi G. J. Choi Y. H. (2012). Production of L -lactic acid from a green microalga, *Hydrodictyon reticulum*, by *Lactobacillus paracasei* LA104 isolated fromthe traditional Korean food, makgeolli. *Biores. Technol.* 110 552–559. 10.1016/j.biortech.2012.01.079 22336740

[B79] Niccolai A. Bigagli E. Biondi N. Rodolfi L. Cinci L. Luceri C. (2017). In vitro toxicity of microalgal and cyanobacterial strains of interest as food source. *J. Appl. Phycol.* 29 199–209. 10.1007/s10811-016-0924-2

[B80] Niccolai A. Shannon E. Abu-Ghannam N. Biondi N. Rodolfi L. Tredici M. R. (2019a). Lactic acid fermentation of *Arthrospira platensis* (spirulina) biomass for probiotic-based products. *J. Appl. Phycol.* 31 1077–1083. 10.1007/s10811-018-1602-3

[B81] Niccolai A. Venturi M. Galli V. Pini N. Rodolfi L. Biondi N. (2019b). Development of new microalgae-based sourdough “crostini”: functional effects of *Arthrospira platensis* (spirulina) addition. *Sci. Rep.-UK.* 9 1–12. 10.1038/s41598-019-55840-1 31857609 PMC6923427

[B82] Niccolai A. Zittelli G. C. Rodolfi L. Biondi N. Tredici M. R. (2019c). Microalgae of interest as food source: biochemical composition and digestibility. *Algal Res.* 42:101617 10.1016/j.algal.2019.101617

[B83] Ogawa J. Kishino S. Ando A. Sugimoto S. Mihara K. Shimizu S. (2005). Production of conjugated fatty acids by lactic acid bacteria. *J. Biosci. Bioeng.* 100 355–364. 10.1263/jbb.100.355 16310724

[B84] Paixao N. Perestrelo R. Marques J. C. Câmara J. S. (2007). Relationship between antioxidant capacity and total phenolic content of red, rosé and white wines. *Food Chem.* 105 204–214. 10.1016/j.foodchem.2007.04.017

[B85] Parada J. L. de Caire G. Z. de Mulé M. C. Z. de Cano M. M. S. (1998). Lactic acid bacteria growth promoters from *Spirulina platensis*. *Int. J. Food. Microbiol.* 45 225–228. 10.1016/S0168-1605(98)00151-2 9927000

[B86] Rajauria G. Jaiswal A. K. Abu-Ghannam N. Gupta S. (2013). Antimicrobial, antioxidant and free radical-scavenging capacity of brown seaweed *Himanthalia elongata* from western coast of Ireland. *J. Food Biochem.* 37 322–335. 10.1111/j.1745-4514.2012.00663.x

[B87] Report I. S. T. I. S. A. N. (1966). ISTISAN (*Istituto Superiore di Sanità*) ISSN 1123-3117 Rapporti ISTISAN 96/34. *Metodi Anal. Util. Controllo Chim. Degli Alimenti.* 1996:265.

[B88] Ricci A. Cirlini M. Calani L. Bernini V. Neviani E. Del Rio D. (2019a). In vitro metabolism of elderberry juice polyphenols by lactic acid bacteria. *Food Chem.* 276 692–699. 10.1016/j.foodchem.2018.10.046 30409649

[B89] Ricci A. Cirlini M. Maoloni A. Del Rio D. Calani L. Bernini V. (2019b). Use of dairy and plant-derived Lactobacilli as starters for cherry juice fermentation. *Nutrients* 11 1–14. 10.3390/nu11020213 30678152 PMC6412669

[B90] Ricci A. Diaz A. B. Caro I. Bernini V. Galaverna G. Lazzi C. (2019c). Orange peels: from by-product to resource through lactic acid fermentation. *J. Sci. Food Agr.* 99 6761–6767. 10.1002/jsfa.9958 31353470

[B91] Riss J. Décordé K. Sutra T. Delage M. Baccou J.-C. Jouy N. (2007). Phycobiliprotein C-phycocyanin from *Spirulina platensis* is powerfully responsible for reducing oxidative stress and NADPH oxidase expression induced by an atherogenic diet in hamsters. *J. Agric. Food Chem.* 55 7962–7967. 10.1021/jf070529g 17696484

[B92] Romay C. Armesto J. Remirez D. González R. Ledon N. García I. (1998). Antioxidant and anti-inflammatory properties of C-phycocyanin from blue-green algae. *Inflamm. Res.* 47 36–41. 10.1007/s000110050256 9495584

[B93] Roy K. R. Arunasree K. M. Reddy N. P. Dheeraj B. Reddy G. V. Reddanna P. (2007). Alteration of mitochondrial membrane potential by *Spirulina platensis* C-phycocyanin induces apoptosis in the doxorubicin-resistant human hepatocellular-carcinoma cell line HepG2. *Biotech. Appl. Biochem.* 47 159–167. 10.1042/BA20060206 17274761

[B94] Sahasrabudhe N. A. Sankpal N. V. (2001). Production of organic acids and metabolites of fungi for food industry. *Appl. Microbiol. Biotechnol.* 47 387–425. 10.1016/S1874-5334(01)80016-2

[B95] Salvetti E. Torriani S. Felis G. E. (2012). The genus *Lactobacillus*: a taxonomic update. *Prob. Antimicrob. Prot.* 4 217–226. 10.1007/s12602-012-9117-8 26782181

[B96] Sangwan N. K. Gupta K. Singh Dhindsa K. (1986). Fatty acidcomposition of developing soybeans. *J. Agric. Food Chem.* 34 415–417. 10.1021/jf00069a008

[B97] Sarada R. M. G. P. Pillai M. G. Ravishankar G. A. (1999). Phycocyanin from *Spirulina* sp: influence of processing of biomass on phycocyanin yield, analysis of efficacy of extraction methods and stability studies on phycocyanin. *Proc. Biochem.* 34 795–801. 10.1016/S0032-9592(98)00153-8

[B98] Savijoki K. Ingmer H. Varmanen P. (2006). Proteolytic systems of lactic acid bacteria. *Appl. Microbial. Biotechnol.* 71 394–406. 10.1007/s00253-006-0427-1 16628446

[B99] Scharf C. Riethdorf S. Ernst H. Engelmann S. Völker U. Hecker M. (1998). Thioredoxin is an essential protein induced by multiple stresses in *Bacillus subtilis*. *J. Bacteriol.* 180 1869–1877. 10.1128/JB.180.7.1869-1877.1998 9537387 PMC107102

[B100] Ścieszka S. Klewicka E. (2019). Algae in food: a general review. *Crit. Rev. Food Sci. Nutr.* 59 3538–3547. 10.1080/10408398.2018.1496319 29999416

[B101] Shabana E. F. Gabr M. A. Moussa H. R. El-Shaer E. A. Ismaiel M. M. (2017). Biochemical composition and antioxidant activities of *Arthrospira* (*Spirulina*) *platensis* in response to gamma irradiation. *Food Chem.* 214 550–555. 10.1016/j.foodchem.2016.07.109 27507509

[B102] Sigler K. Chaloupka J. Brozmanova J. Stadler N. Hofer M. (1999). Oxidative stress in microorganisms - I - Microbial vs. higher cells - Damage and defenses in relation to cell aging and death. *Folia Microbiol.* 44 587–624. 10.1007/BF02825650 11097021

[B103] Slatnar A. Jakopic J. Stampar F. Veberic R. Jamnik P. (2012). The effect of bioactive compounds on *in vitro* and *in vivo* antioxidant activity of different berry juices. *PLoS One* 7:e47880. 10.1371/journal.pone.0047880 23110118 PMC3479138

[B104] Smid E. J. Kleerebezem M. (2014). Production of aroma compounds in lactic fermentations. *Ann. Rev. Food Sci. Technol.* 5 313–326. 10.1146/annurev-food-030713-092339 24580073

[B105] Sozer N. Melama L. Silbir S. Rizzello C. G. Flander L. Poutanen K. (2019). Lactic acid fermentation as a pre-treatment process for faba bean flour and its effect on textural, structural and nutritional properties of protein-enriched gluten-free faba bean breads. *Foods* 8:431. 10.3390/foods8100431 31546650 PMC6836149

[B106] Suzuki Y. Kosaka M. Shindo K. Kawasumi T. Kimoto-Nira H. Suzuki C. (2013). Identification of antioxidants produced by *Lactobacillus plantarum*. *Biosci. Biotech. Bochem.* 77 1299–1302. 10.1271/bbb.121006 23748762

[B107] Tanaka T. Hoshina M. Tanabe S. Sakai K. Ohtsubo S. Taniguchi M. (2006). Production of D-lactic acid from defatted rice bran by simultaneous saccharification and fermentation. *Biores. Technol.* 97 211–217. 10.1016/j.biortech.2005.02.025 16171677

[B108] Thompson H. O. Önning G. Holmgren K. Strandler H. S. Hultberg M. (2020). Fermentation of cauliflower and white beans with *Lactobacillus plantarum*–impact on levels of riboflavin, folate, vitamin B 12, and amino acid composition. *Plant Food. Hum. Nutr.* 75 236–242. 10.1007/s11130-020-00806-2 32144644 PMC7266841

[B109] Tibbetts S. M. Santosh P. Lall S. P. Milley J. E. (2012). “Protein quality of microalgae based on in vitro digestibility and amino acid profile,” in *Annual Conference of the International Society for Nutraceuticals and Functional Foods*, Hawaii, 2–6.

[B110] Tredici M. R. Rodolfi L. Biondi N. Bassi N. Sampietro G. (2016). Techno-economic analysis of microalgal biomass production in a 1-ha Green Wall Panel (GWP^®^) plant. *Algal Res.* 19 253–263. 10.1016/j.algal.2016.09.005

[B111] Tsakalidou E. Anastasiou R. Vandenberghe I. Van Beeumen J. Kalantzopoulos G. (1999). Cell-wall-bound proteinase of *Lactobacillus delbrueckii* subsp. lactis ACA-DC 178: characterization and specificity for β-casein. *Appl. Environ. Microbiol.* 65 2035–2040. 10223997 10.1128/aem.65.5.2035-2040.1999 PMC91294

[B112] Uchida M. Miyoshi T. (2013). Algal fermentation-the seed for a new fermentation industry of foods and related products. *Japan Agr. Res. Quart* 47 53–63. 10.6090/jarq.47.53

[B113] Urbach G. (1997). The flavour of milk and dairy products: II. Cheese: contribution of volatile compounds. *Int. J. Dairy Tech.* 50 79–89. 10.1111/j.1471-0307.1997.tb01743.x

[B114] Vanderhoof J. A. Young R. J. (1998). Use of probiotics in childhood gastrointestinal disorders. *J. Ped. Gastr. Nutr.* 27 323–332. 10.1097/00005176-199809000-00011 9740206

[B115] Varga L. Szigeti J. Kovács R. Földes T. Buti S. (2002). Influence of a *Spirulina platensis* biomass on the microflora of fermented ABT milks during storage (R1). *J. Dairy Sci.* 85 1031–1038. 10.3168/jds.S0022-0302(02)74163-5 12086036

[B116] Wang X. Shao C. Liu L. Guo X. Xu Y. Lü X. (2017). Optimization, partial characterization and antioxidant activity of an exopolysaccharide from *Lactobacillus plantarum* KX041. *Int. J. Biol. Macromol.* 103 1173–1184. 10.1016/j.ijbiomac.2017.05.118 28551435

[B117] Wolfe K. L. Liu R. H. (2007). Cellular antioxidant activity (CAA) assay for assessing antioxidants, foods, and dietary supplements. *J. Agric. Food Chem.* 55 8896–8907. 10.1021/jf0715166 17902627

[B118] Wu H. L. Wang G. H. Xiang W. Z. Li T. He H. (2016). Stability and antioxidant activity of food-grade phycocyanin isolated from *Spirulina platensis*. *Int. J. Food Prop.* 19 2349–2362. 10.1080/10942912.2015.1038564

[B119] Yadav H. Jain S. Sinha P. R. (2007). Production of free fatty acids and conjugated linoleic acid in probiotic dahi containing *Lactobacillus acidophilus* and *Lactobacillus casei* during fermentation and storage. *Int. Dairy J.* 17 1006–1010. 10.1016/j.idairyj.2006.12.003

[B120] Yamaguchi S. K. F. Moreira J. B. Costa J. A. V. de Souza C. K. Bertoli S. L. Carvalho L. F. D. (2019). Evaluation of Adding *Spirulina* to Freeze-Dried Yogurts Before Fermentation and After Freeze-Drying. *Ind. Biotechnol.* 15 89–94. 10.1089/ind.2018.0030

[B121] Yoon K. Y. Woodams E. E. Hang Y. D. (2006). Production of probiotic cabbage juice by lactic acid bacteria. *Bioresour. Technol.* 97 1427–1430. 10.1016/j.biortech.2005.06.018 16125381

[B122] Zakrajsek T. Raspor P. Jamnik P. (2011). *Saccharomyces cerevisiae* in the stationary phase as a model organism - characterization at cellular and proteome level. *J. Proteomics.* 74 2837–2845. 10.1016/j.jprot.2011.06.026 21782986

[B123] Zarrouk C. (1966). *Contribution a l’etude d’une Cyanophycee. Influence de Divers Facteurs Physiques et Chimiques sur la croissance et la photosynthese de Spirulina mixima.* Thesis, University of Paris, France.

[B124] Zhao D. Shah N. (2016). Lactic acid bacterial fermentation modified phenolic composition in tea extracts and enhanced their antioxidant activity and cellular uptake of phenolic compounds following in vitro digestion. *J. Func. Foods* 20 182–194. 10.1016/j.jff.2015.10.033

